# Payment problems and suicide: life under financial strain

**DOI:** 10.1177/14034948241312375

**Published:** 2025-03-31

**Authors:** Carla l. Hughes, Åsmund Hermansen

**Affiliations:** 1 National Centre for Suicide Research and Prevention (NSSF), University of Oslo (UiO), Oslo, Norway; 2 Department of Social Work, Child Welfare, and Social Policy, OsloMet, Oslo Metropolitan University, Oslo, Norway

**Keywords:** Payment problems, debt, suicide, stress

## Abstract

**Aims:**

Suicide deaths are often linked to impulsivity during moments of crisis, such as financial difficulties, relationship breakdowns and poor health. For individuals experiencing financial problems, risk factors for suicide can commonly include circumstances surrounding payment problems, including unemployment, divorce, low education and low income mediated by debt.

**Methods:**

In this study, we investigate the impact of payment problems on suicide in Norway using a spline-based parametric survival analysis, with suicide as the outcome variable. With access to high quality Norwegian register data and unique information on payment problems, defined as deductions in wages or benefits, we investigated suicide amongst the entire adult Norwegian population over an 11-year period (2008–2018).

**Results:**

We found that the prevalence of payment problems among the Norwegian population between 2009 and 2018 was associated with a higher risk of suicide for both males and females. Despite the greater proportion of suicide occurring amongst males both globally and in Norway, we found that women experiencing financial hardship had a relatively higher suicide risk when adjusted for demographic variables than their male counterparts.

**Conclusions:**

**These findings highlight the need for stronger protections for individuals struggling with financial difficulties and emphasise the importance of further research on the relationship between payment problems and suicide, with the aim of informing and enhancing national suicide prevention strategies.**

## Background

Death by suicide refers to the act of taking one’s own life. Across the globe, more than 700,000 people die by suicide each year, amounting to one person every 40 seconds [1]. For good reason, the World Health Organisation (WHO) has characterised suicide as a serious universal public health issue [2]. However, death by suicide is a complicated phenomenon whereby intent is difficult to determine. However, it is possible to observe environmental and personal ‘triggers’ that may increase risk of suicide.

The precise aetiology of suicidal behaviours is difficult to determine, although it is possible to demonstrate structural factors involved in the increased likelihood of suicide. These factors include poverty, unmanageable debts, financial problems and other stressful life events surrounding economic recession [3–5]. Literature indicates that individuals facing such struggles may experience amplified stress, shame, loneliness and hopelessness [16–18], all of which contribute to a heightened risk of suicide.

In a 2021 report, WHO determined that several suicide deaths were linked directly to impulsivity during moments of crisis, including financial problems, relationship breakdowns and poor health [2]. Life events such as unemployment [6–8], divorce [9,10], low level education [11] and low income, particularly when mediated by debt, are recognised as situational triggers that increase suicidal ideation and risk [2,13]. These personal challenges may lead individuals to experience overwhelming emotional strain and increase suicidal behaviours.

## Suicide and payment problems

The presence of financial strain and debt has been linked to heightened suicide risk. Relevant economic risk factors for suicide may include poverty, unmanageable debts, financial problems and other stressful life events surrounding economic recession. Individuals dealing with payment problems often face significant levels of social stress, which contributes to feelings of hopelessness, isolation and shame [6–8]. This is further aggravated when one’s access to fulfilling social, physical and healthcare activities is restricted. Speculatively, the level of risk may be proportional to the severity and duration of financial problems [7,9]. In essence, by living narrowly within one’s basic needs, and scarcely meeting the margins, many people have exhausted their ability to live a healthy and fulfilled life, ultimately leading to social isolation, deliberate self-harm or suicide [6,10,11].

Unemployment, particularly under economic recession, is suggested to be a high-level risk factor for suicide [12]. More specifically, abrupt loss of employment and the consequential financial issues that are synonymous with economic recession are suggested to put one at greatest risk of suicide [13]. Consequently, the amalgamation of risk factors over time assists in understanding the life-lived of a person struggling financially as opposed to at one moment in time, which our study aims to explore.

## Social stress process model

The social stress process model asserts a relationship between social disadvantages and health. According to Pearlin et al. [14], the model includes three domains: stress sources, stress outcomes and stress mediators. In the context of this study, stress sources include economic hardship. These sources serve to explain stress outcomes such as reduced personal accomplishment and or emotional exhaustion [15,16], both of which indicate an increased risk of suicide. Furthermore, lack of social support or coping mechanisms exacerbates vulnerability to these stressors, reinforcing the negative impacts of financial hardship.

In Norway, the Norwegian Institute of Public Health (FHI) report that over 650 people die of suicide each year in Norway, 2 out of 3 of which are male [17]. Accordingly, approximately 13.3 per 100,000 died by suicide between 2000 and 2022 [18]. For individuals experiencing financial difficulties, Norway’s statistics reflect global trends, with socioeconomic factors such as low education and unemployment associated with increased suicide risk. Remaining employed and higher educational qualifications are reported to be advantageous protective factors against financial hardship [19]. According to European studies, Norwegian men with only secondary education are at higher suicide risk compared with those with advanced education, whereas lower education levels offer a slight protective factor for women [11].

The aim of our study is to investigate the impact of payment problems on rates of suicide within Norway. With access to high quality Norwegian register data and unique information on payment problems, defined as deductions in wages or benefits, we investigate suicide in the adult Norwegian population over an 11-year period (2008–2018). By using a spline-based parametric survival analysis, we aim to understand how financial hardship affects the risk of suicide. Additionally, we explore the relationship between societal factors, such as education, as well as the role of social support systems, such as marital status, in potentially mitigating suicide risk.

To our knowledge, this is the first study investigating the impact of payments problems on the likelihood of suicide for the whole adult Norwegian population. Using register data, we are able to determine to what extent payment problems impact the likelihood of suicide in Norway.

## Methods

### Data and variables

We used Norwegian register data from Statistics Norway (SSB) and the Norwegian Institute of Public Health (FHI), including Norwegian Cause of Death Registry (DÅR). The population sample was restricted to individuals aged 18 years and older in the years 2009–2018, with the intention on capturing those within working age who are also at highest risk of both suicide and payment problems.

### Dependent variable

Our outcome variable *Suicide* is categorised in the Norwegian Cause of Death Registry (DÅR) in accordance with The International Statistical Classification of Diseases and Related Health Problems, 10th Revision, (ICD-10) as an external cause of morbidity and mortality and is further defined as death by intentional self-harm (X60-X84).

### Independent and control variables

Using Norwegian register data, we measured *payment problems* between the years 2009 and 2018, by registration of deductions in income or benefits. Deduction in wages or benefits is the most common form of debt enforcement in Norway. The deductions are measured monthly and accordingly we can identify financial strain monthly over the 11-year period included in this paper.

In this study we also controlled for age, sex, level of education, marital status, immigrant status and household income. All the control variables were measured in 2009. Age is included in the analysis as a continues variable, sex is measured as a dummy variable and education separates those who have finished a university education at the bachelor level or higher and those with less education. For marital status, we separated married, divorced, widow or widower and single. Immigrant status separates those who were born in Norway to two Norwegian parents and those who were not. Household income is measured using equivalized disposable household income as defined by Eurostat and calculated as the total household income after deduction of taxes and social contributions divided by the number of ‘equivalent adults’ reflecting the size and the age composition of the household by weighting all members of the household.

## Analytical strategy

We first performed a descriptive analysis of those who died by suicide and those who did not, including information on dedications in income or benefits, sex, age, immigration status, level of education, marital status and household income. Since we were interested in looking at survival time for those who had a deduction in wages or benefits and those who did not, using Cox regression was an obvious choice. However, testing the proportional hazard (PH) assumption showed that the hazard was not constant over time for those who experienced deductions and those who did not. Accordingly, we employed a spline-based parametric survival model using the stpm command in Stata version 16 [20]. Following Royston and Parmar [21], we selected the spline part of the model by minimizing the Akaike Information Criterion (AIC). Furthermore, we used the AIC to choose the scale for the model. Comparing the AIC for a parametric proportional hazards model and a parametric proportional odds model showed that an odds model with six knots (df = 6) resulted in the lowest AIC. We then examined the relationship between deductions and suicide death, controlling for sex, age, immigration status, level of education, marital status and household income.

## Results

The results presented in Table I shows that 0.14 percent of Norwegian adults, or 5013 individuals, died by suicide between 2009 and 2018. Among those who experienced a deduction in wages or benefits, 0.29 percent committed suicide compared with 0.12 percent among those who did not experience a deduction. These results also demonstrate variations in suicide rates based on sex, immigration status, education level, marital status and household income.

**Table I.** Descriptive statistics.

	*Suicide*	
Variables	(=1)	(=0)
	5013 (0.14%)	3,616,789 (99.86%)
Deduction in income or benefits		
Yes	1185 (0.29%)	410,600 (99.71%)
No	3828 (0.12%)	3,206,198 (99.88%)
Sex		
Male	3507 (0.20%)	1,782,475 (99.80%)
Female	1506 (0.08%)	1,834,323 (99.92%)
Age, years (mean)	45.8	48.7
Immigration status		
Born in Norway by two Norwegian parents	4416 (0.14%)	3,133,638 (99.86%)
Others	597 (0.12%)	483,160 (99.88%)
Level of education		
Higher education	977 (0.10%)	1,025,683 (99.90%)
High school or less educated	4036 (0.16%)	2,591,115 (99.84%)
Marital status		
Married	1516 (0.09%)	1,691,595 (99.91%)
Divorced	922 (0.22%)	413,578 (99.78%)
Widow or widower	172 (0.07%)	252,236 (99.93%)
Single	2,403 (0.19%)	1,259,389 (99.81%)
Household income		
Equalized household income >60 percent of median	4572 (0.13%)	3,518,812 (99.87%)
Equalized household income <60 percent of median	441 (0.45%)	97,986 (99.55%)

**Figure 1.**
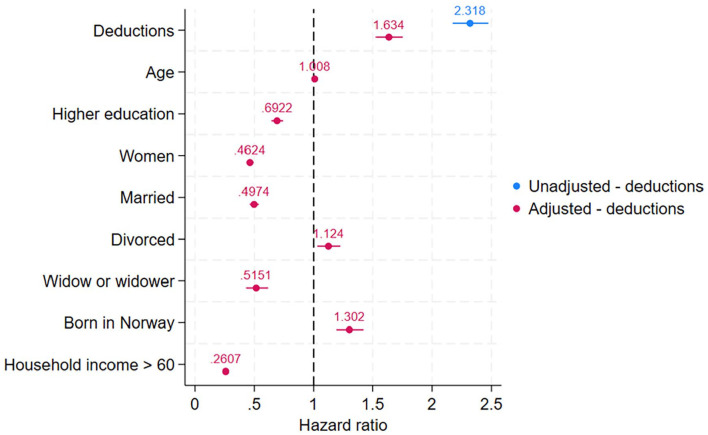
Results using a spline-based parametric survival model using suicide as dependent variable (2009–2018). *N* = 3,621,811.

The unadjusted results show that the risk of suicide increase by 2.3 times for each deduction a Norwegian adult experienced during 2009 to 2018 (Figure 1). After controlling for sex, age, immigration status, level of education, marital status and household income, experiencing a deduction in wages or benefits increased the risk of suicide by 1.6 times.

**Figure 2.**
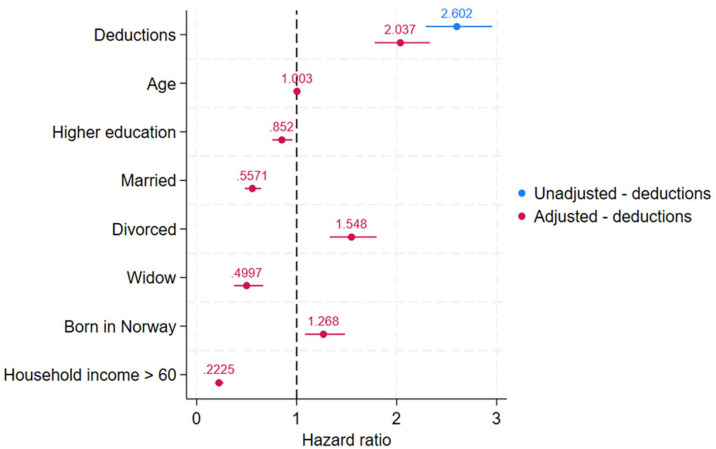
Results using a spline-based parametric survival model using suicide as dependent variable—including only women (2009–2018). *N* = 1,835,829.

The unadjusted results show that, for women, the risk of suicide increased by 2.6 times for those experiencing a deduction during 2009–2018 (Figure 2). After adjusting for age, immigration status, level of education, marital status and household income, women experiencing a deduction in wages or benefits have a 2.0 times elevated risk of suicide.

**Figure 3.**
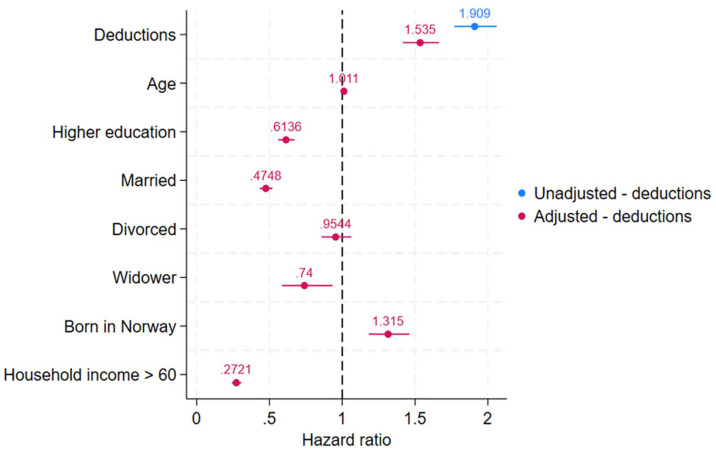
Results using a spline-based parametric survival model using suicide as dependent variable—including only men (2009–2018). *N* = 1,785,982.

The unadjusted results for men show that for those experiencing a deduction during 2009–2018 the risk of suicide increased by 1.9 times (Figure 3). After adjusting for age, immigration status, level of education, marital status and household income, for each deduction experienced during 2009–2018, Norwegian men have a 1.5 times elevated risk for suicide.

## Discussion and implications

In this study, we aimed to investigate the impact of payment problems on the risk of suicide in the Norwegian adult population from 2009 to 2018. Our research questions focused on examining whether payment problems serves as a significant risk factor for suicide in both men and women, and investigated whether demographic variables such as sex, age, marital status and household income influence this relationship.

The results of our study reveal an increased risk for suicide for both men and women experiencing payment problems. Despite the greater proportion of suicide occurring amongst men, both nationally and globally, we found that women experiencing financial hardship had a relatively higher suicide risk even after adjusting for demographic variables. This novel finding in the Norwegian population suggests that greater protection is required for individuals coping with financial issues and highlights a critical area for improvement in gender-sensitive health and social interventions.

The Norwegian welfare state offers a range of social protections to all residents, regardless of immigration background, most notably affordable healthcare, free education and attractive pension schemes. Correspondingly, Norway has some of the highest incomes and lowest income inequalities in the world. However, as supported by our research, this does not shield all residents from financial strain throughout their lifetime, and payment problems still occur and the associated outcome of suicide is increased under such economic hardships. Indeed, the risk of payment problems and suicidal behaviours in Norway are speculated to be impacted by several individual components of the Norwegian welfare system, which may be limited by flaws [5]. Addressing this requires coordinated efforts across welfare administration, creditors and healthcare providers to strengthen financial support and prevent suicide and provide a safety net to aid those in need.

In line with the social stress process model, our findings also demonstrate that marital status was strongly associated with suicide risk and payment problems [22], particularly amongst those who were divorced. For individuals who are divorced, the absence of close support increases their vulnerability when facing financial hardship. In further accordance with the stress process model, coping with life strains includes having access to individuals, groups or organisations from which one is able to learn, and sharing within groups of which they are a member [14], and marital status implicates a group or individual that one has access to.

## Strengths and limitations

Our study has several strengths, including its use of comprehensive Norwegian registry data covering the entire adult population throughout the study period. The register data enabled a robust analysis of payment problems and suicide risk with adjustment for demographic variables. However, our study is subject to certain limitations. First, although registry data allows for wide ranging insights into the adult population as a whole, it may lack specificity on an individual basis to capture individual subjective experiences of financial stress. Second, our study is specific to Norway, which may reduce generalisability to lower income countries with significantly different social support systems.

## Conclusion

Suicide remains a multifaceted social problem with several proposed solutions that require diverse prevention strategies. The WHO emphasises that prevention of suicide can be implemented with evidence-based and low-cost interventions [2]. In 1995, Norway became the second country in the world, after Finland, to launch a suicide prevention model entitled ‘The Norwegian chain-of-care model’. Aspects of the original model have continued to be updated and have been implemented by both the Norwegian Directorate of Health and the Norwegian government since initiation [3]. The report ‘Handlingsplan for forebygging av selvmord’ (Action plan for prevention of suicide) written as part of the national plan for suicide prevention, demonstrates methods by which suicide can be prevented in Norway, as well as highlighting several risk factors for suicide including mental health issues and societal factors. Whereas the prevention model identifies debt as a risk factor and addresses financial risk factors linked to gambling and gaming addiction, it does not fully account for other forms of financial hardship, such as debt unrelated to gambling. Our study highlights that payment problems, particularly for women, are a significant suicide risk factor even when unrelated to gambling. This indicates a substantial gap in the national prevention plan, which overlooks certain high-risk populations.

To extend the identification of stress as proposed by Pearlin et al. [14], we advocate for improved identification and support for individuals with payment problems, as they may be especially vulnerable to suicide and suicidal behaviours.
